# Philadelphia County Medical Society

**Published:** 1881-05

**Authors:** 


### Reports of Societies.

---

### PHILADELPHIA COUNTY MEDICAL SOCIETY.

At a conversational meeting of the Society, held at the Hall of the College of Physicians, Philadelphia, January 12, 1881, Dr. Albert H. Smith, President, in the chair, Dr. Frank Woodbury read a paper on "Some Clinical Phases of Poisoning by Alcohol."

*Discussion.*—Dr. E. T. Bruen noted the similarity between the brain symptoms of chronic alcoholism and those of ordinary chronic meningitis, and inquired regarding the differential diagnosis. He had observed in many cases of alcoholism irregular rises in temperature, and after death thickening of the membranes, adhesion, and other evidences of inflammation

Dr. R. S. Huidekoper related a case of idiosyncrasy in regard to the effects of alcohol. While on a boating excursion a young man took within fifteen minutes a couple of drinks of whisky amounting to less than two ounces in all. In a short time his face became flushed, and suddenly grew very pale; he then fell into a state of complete alcoholic coma. It was nearly six hours before he regained consciousness. It was subsequently ascertained that this gentleman had suffered on previous occasions from similar small quantities of alcohol.

Dr. John B. Roberts said that the physician has better opportunities of recognizing the effect of alcohol, and of studying the relations of cause and effect in alcoholism, than in many other diseases. The question whether or not alcohol is a true food is a very important one. Dr. Parkes, in his report of the issue of a spirit ration in the Ashantee campaign, arrived at the conclusion that for forced marches of short duration, especially at night, the troops did better on a rum ration, but that their energies were depressed the next day and they were correspondingly incapacitated from sustained effort; but on an extra ration of beef tea their efforts could be kept up to the maximum for several days, with less fatigue.

Delirium tremens is not always due to deprivation of alcohol, but in many cases it is due to cerebral anæmia from want of food. It is not, therefore, to be regarded as an indication to return to alcohol-drinking so much as it is to administer nutritious food. In many cases tonics can be substituted for spirits, and the cases recover as well or even more readily than with whisky.

Dr. Charles K. Mills said that he regarded the remarks of the lecturer upon the differential diagnosis of acute alcohol-poisoning as of considerable importance. His own experience had been that it was very difficult to make an exact diagnosis of alcoholic coma. The points mentioned in the paper were good, but they would be insufficient sometimes to decide the character of a case. Hemiplegia, for instance, is not always present in apoplexy. He had seen a case lately where a large hemorrhage had broken down part of the cerebral hemisphere and the septum between the ventricles, both sides of the body at the time of examination being about equally helpless. In another case where coma was present it was difficult to decide whether it was uræmic, alcoholic or apoplectic. The man was brought into the hospital ward insensible. Respirations were very slow—indeed, only about one-third of what they should have been. The skin was so cold that surface-temperature could not be taken with ordinary instruments, and in the rectum the temperature was under 90°. Complete paralysis existed, not hemiplegia. There was no odor of alcohol on the breath ; no appearance of injury to the head; no swelling of the feet and legs. Albumen was present in the urine. Upon making the autopsy, cerebral œdema was found ; a large amount of fluid was present between the pia mater and entire convexity of the brain ; it was also increased in the ventricles. The kidneys were degenerated. It was a case of uræmia.

While in exceptional cases a positive diagnosis could not be made, still the presence of an alconolic odor of the breath, the chemical examination of the urine, the surface temperature, and the paralytic symptoms would largely guide us to a correct conclusion.

In reference to the treatment of acute and chronic alcoholism he endorsed the remarks of the lecturer. He had found in coma and threatened callapse that the expedient of administering belladonna or atropia hypodermically might be a most important one, and it was one that was not as largely used as it should be. It is especially valuable when death is threatened by respiratory paralysis. He had used atropia in this manner, combined with electricity, as a peripheral stimulant. He mentioned the following case, although not one of alcoholism. A patient had been almost constantly vomiting for nearly three weeks, with extreme depression of respiration, the movements being reduced to four or five in the minute. Under the use of electricity to the limbs and atropia hypodermically, she rallied from this condition and lived four or five weeks, when she had another attack, in which she died. It was subsequently found that she had a cerebellar tumor. In nearly all the cases that he had examined of chronic alcoholism he had noticed changes inflammatory in character, especially in the pia mater; a peculiar opacity was usually present; but the pathological condition was not identical with that of meningitis from ordinary causes. He had also seen pachymeningitis in drunkards.

Dr. W. H. Parish, as illustrating the difficulties of diagnosis, referred to a case which had occurred at the Philadelphia Hospital. A man, who had been several times previously under treatment for a similar condition, was admitted into the drunk ward, moderately intoxicated. After some hours' rest he apparently recovered, and was employed about the institution for several days One night he was taken with what was supposed to be mania dependent upon alcoholism, and he was treated for several days for delirium tremens. His death occurred shortly afterwards, and at the post-mortem examination the blade of a pen-knife was found imbedded in the middle of the brain, having entered through the squamous portion of the temporal bone; the whole blade was there; it had led to extensive abcess, meningitis, and cerebritis. The symptoms throughout had been apparently those of delirium tremens.

Dr. Bruen said that he had seen cases in which he should have liked to have something upon which to base a decided opinion as to the existence of meningitis as a complication; for in some cases meningitis had been found after death, although he had not been able to distinguish the symptoms from those of ordinary delirium tremens during life.

Dr. Woodbury, in closing the discussion, said that the clinical phases of alcohol-poisoning offered an interesting and important topic for the practitioner, on account of the frequency of their occurence and their usually urgent character. He had been surprised upon reviewing the proceedings, that this subject had not within the last few years been brought up before the Society for discussion. On account of its comprehensive character he had only been able to sketch, and with rather a free hand, some of the rough outlines of the more prominent considerations springing out of the subject which he had submitted in the paper of the evening. In regard to the diagnosis of chronic meningitis he agreed with a previous speaker, that in simple cases occurring independently of alcoholism due attention to the symptoms would be sufficient to distinguish the case from ordinary delirium tremens. The previous history—of syphilis, sunstroke, blows upon the skull, or, rarely, rheumatism, with localized pain in the head of severe character, exacerbations of fever and delirium, with vomiting, but with onset less sudden than delirium tremens—may serve to distinguish such cases. When the conditions coexist, the use of the surface thermometer to the scalp would probably indicate the seat of the inflammatory process and aid in the diagnosis. If the patient complains of intense headache, and there is a decided febrile movement, frequent vomiting, and irregularly contracted pupils, the view of the coexistence of meningitis is tenable, and is at least highly probable. The fact that this complication is a frequent one has already been dwelt upon.

In concluding, he stated that although a man under the influence of a full dose of alcohol would generally succeed in sleeping off its effects, if kept warm, yet he felt that science—which shows alcohol to be a respiratory poison and capable of destroying life—protests against allowing a man in a condition of coma to sleep with an unknown amount of alcohol in his stomach waiting to be absorbed. When received into a hospital or station-house, such a patient should have his stomach emptied by an emetic or by the pump, and have a pint of hot coffee administered before allowing him to sleep; at the same time the skull should be carefully examined for injuries which may require prompt surgical attention.

